# Costs and Cost-Effectiveness of User-Testing of Health Professionals’ Guidelines to Reduce the Frequency of Intravenous Medicines Administration Errors by Nurses in the United Kingdom: A Probabilistic Model Based on Voriconazole Administration

**DOI:** 10.1007/s40258-021-00675-z

**Published:** 2021-08-17

**Authors:** Matthew D. Jones, Bryony Dean Franklin, D. K. Raynor, Howard Thom, Margaret C. Watson, Rebecca Kandiyali

**Affiliations:** 1 Department of Pharmacy and Pharmacology, University of Bath, Bath, BA2 7AY UK; 2 UCL School of Pharmacy, London, UK; 3 Pharmacy Department, Centre for Medication Safety and Service Quality, Imperial College Healthcare NHS Trust, London, UK; 4 School of Healthcare, University of Leeds, Leeds, UK; 5 Luto Research, Leeds, UK; 6 Bristol Medical School, University of Bristol, Bristol, UK; 7 Strathclyde Institute of Pharmacy and Biomedical Sciences, University of Strathclyde, Glasgow, UK

## Abstract

**Aim:**

In the UK, injectable medicines are often prepared and administered by nurses following the Injectable Medicines Guide (IMG). Our earlier study confirmed a higher frequency of correct administration with user-tested versus standard IMG guidelines. This current study aimed to model the cost-effectiveness of user-testing.

**Methods:**

The costs and cost-effectiveness of user-testing were explored by modifying an existing probabilistic decision-analytic model. The adapted model considered administration of intravenous voriconazole to hospital inpatients by nurses. It included 11 error types, their probability of detection and level of harm. Model inputs (including costs) were derived from our previous study and other published data. Monte Carlo simulation using 20,000 samples (sufficient for convergence) was performed with a 5-year time horizon from the perspective of the 121 NHS trusts and health boards that use the IMG. Sensitivity analyses were undertaken for the risk of a medication error and other sources of uncertainty.

**Results:**

The net monetary benefit at £20,000/quality-adjusted life year was £3,190,064 (95% credible interval (CrI): −346,709 to 8,480,665), favouring user-testing with a 96% chance of cost-effectiveness. Incremental cost-savings were £240,943 (95% CrI 43,527–491,576), also favouring user-tested guidelines with a 99% chance of cost-saving. The total user testing cost was £6317 (95% CrI 6012–6627). These findings were robust to assumptions about a range of input parameters, but greater uncertainty was seen with a lower medication error risk.

**Conclusions:**

User-testing of injectable medicines guidelines is a low-cost intervention that is highly likely to be cost-effective, especially for high-risk medicines.

**Supplementary Information:**

The online version contains supplementary material available at 10.1007/s40258-021-00675-z.

## Key Points for Decision Makers

**Table:** 

User-testing injectable medicines guidelines for nurses reduces the number of medication errors made when giving intravenous medicines to hospital inpatients.
As a result, the cost of the user-testing process is likely to be outweighed by the increased health of patients and reduced health service costs for treating the harm caused by medication errors.
Therefore, user-testing of guidelines for injectable medicines is a relatively low-cost and cost-effective intervention.

## Introduction

Medication errors are a leading cause of avoidable patient harm, costing an estimated $42 billion per annum worldwide [1]. Each year in the USA, there are an estimated 1.2 million hospitalisations affected by an injectable medicine error, increasing costs by $2.7–5.1 billion [2]. Errors are particularly common with intravenous medicines, with 35–48% of doses containing at least one error [3, 4]. In UK observational studies, 22–74% of adult intravenous medication errors have been classified as having moderate to severe potential consequences [5–7]. In a point prevalence study, 90% of intravenous medication errors were classified as unlikely to cause harm, but 9% were likely to require increased monitoring or intervention and 0.4% to cause temporary harm [8]. Of the many potential causes of patient safety incidents, poor quality written guidance for health professionals has received little attention from researchers [9, 10].

In the UK, nurses prepare and administer most intravenous doses on hospital wards. In over 120 hospital organisations, they use the NHS (National Health Service) Injectable Medicines Guide (IMG) website as a source of written information on how to do this for each dose. Two linked studies have shown that nurses using a user-tested version of the IMG were more likely to correctly prepare and administer an intravenous medicine, and did this more quickly, than nurses using the current version [11, 12]. User-testing is a process based on iterative rounds of interviews with users to discover where they misunderstand a written document and to test potential improvements [11]. This process was applied to the current IMG guideline for voriconazole, which was chosen as it is a high-risk medicine requiring multiple stages of preparation [11]. This resulted in a user-tested voriconazole guideline. In our in situ simulation experiment (hereafter described as the “clinical study”), 273 hospital nurses were randomised to use existing or user-tested versions of the guidelines to administer an infusion of voriconazole to a manikin arm while working on their usual ward [12]. Direct observation was used to identify 11 different types of medication error (Table A of the Online Supplemental Material), which were defined as any deviation from the simulated medication order, the hospital’s policies or the IMG. The potential level of harm for each observed error was assigned using a validated method by seven physicians, pharmacists and nurses who independently scored each error on a scale from 0 (no harm) to 10 (death). A mean score of < 3 was classified as minor harm, a mean score of 3–7 was moderate harm and a mean score > 7 was severe harm. More infusions were completed without any IMG-related errors with the user-tested guidelines (*n* = 67, 48%) than with current guidelines (*n* = 26, 20%) (risk ratio 2.46; 95% confidence interval (CI) 1.68–3.60). As medication errors can cause harm and thus increase health service costs [13], there is potential for the costs of user-testing to be offset by cost-savings resulting from fewer errors. However, the clinical study did not consider cost-effectiveness, so it is unclear whether user-testing of IMG guidelines should be introduced.

Economic decision analytic models can synthesise and extrapolate evidence from randomised trials and other sources to compare the expected costs and consequences of different decision options [14]. The aim of this study, therefore, was to build an economic decision model to assess the cost-effectiveness of user-testing injectable medicines guidelines. To date, studies of the economic impact of medication errors have largely considered their effects on health service costs [15, 16]. Similarly, many economic evaluations of interventions to prevent medication errors have considered only the cost of the intervention and the health services costs avoided by reducing errors [17, 18]. Few of these studies have also considered patients’ quality of life, although this has been recently recommended [15]. Such an approach will enable a more complete assessment of the effects of error-prevention interventions and enable direct comparison with the cost-effectiveness of other interventions. It may also increase uncertainty due to the limitations of the available data on the effects of errors on quality of life. This study therefore had two specific objectives: (i) to investigate the cost impact of user-testing, by comparing the costs of the user-testing process with the reduced costs resulting from fewer medication errors and faster drug preparation, and (ii) to investigate the cost-effectiveness of user-testing in terms of net monetary benefits (derived from quality-adjusted life years; QALYs).

## Methods

There are numerous types of medication error and therefore many potential pathways for the development of harmful effects. Economic models of interventions to reduce medication errors therefore usually consider general categories of harm severity, rather than specific pathways. To do this, several parameters must be determined, including the number of errors with and without the intervention, the likelihood of detection of errors before administration, the level of harm produced, the cost of treating such adverse effects, and the intervention costs. In addition, to achieve our second objective, we also needed to consider the effects of harmful errors of different levels of severity on patients’ quality of life.

Three previous economic models of interventions to reduce medication errors were identified (Table 1). The probabilistic decision tree model developed by Karnon et al. [19] most closely meets the requirements of our objectives, as it relates to errors involving medication use in a UK hospital, considers quality of life, and classifies the harm that may follow a medication error into similar categories to those used in our previous clinical study. We therefore chose to adapt this model to compare the costs and cost-effectiveness of user-tested injectable medicines guidelines compared with current guidelines.

**Table 1:** Summary of previous economic models of interventions to reduce medication errors

First author and year	Study population and setting	Modelling method	Categorisation of harm from errors	Outcome measures
Karnon, (2008) [21]	Inpatients of a 400-bed acute hospital in the UK	Decision tree	No harm, minor, moderate and severe/life-threatening harm	Incremental costs of correcting an error detected before administration, and treating the adverse effects of an error including extra tests, treatments and hospital length of stay Monetary valuation of QoL reduction following a harmful error
Lahue, (2012) [2]	Hospital inpatients receiving injectable medicines in the USA	Not specifically named, but effectively a decision tree	Harm and no harm	Incremental cost of a harmful medication error
Samp, (2014) [22]	Primary, secondary and tertiary care patients in the USA	Decision tree	8 categories from combining: temporary, permanent or no harm; hospitalisation; treatment required; death	Incremental costs of the error, including monitoring and those arising from permanent harm

*QoL* quality of life

We followed the Consolidated Health Economic Evaluation Reports Standards [20].

### Perspective, Population and Setting

The IMG is currently used by nurses preparing medicines in the hospitals of 121 NHS trusts and health boards throughout the UK (Robin Burfield, NHS Wales Informatics Service, personal communication, 30 July 2019). These organisations also share resources to maintain and develop the guide. We therefore performed analysis from the perspective of the NHS payer. The population was hospital inpatients receiving intravenous voriconazole in all 121 hospital trusts and health boards within our perspective (see Sect. 2.6.4).

### Intervention and Comparator

The intervention was the user-testing process to revise the design of the IMG [11]. This involved four pilot interviews followed by three iterative rounds of interviews each with ten nurses, which identified problems finding and understanding information. Each round was followed by revisions to the IMG to resolve problems. We assumed that user-tested guidelines would be introduced by updating the IMG website, with the same cost as provision of the current version of the IMG (Sect. 2.7.3). We compared use of the user-tested IMG with the current version of the IMG.

### Model Outcomes

All outcomes were calculated across the time horizon (Sect. 2.4) for the whole cohort of patients from 121 NHS trusts and health boards within our perspective (Sect. 2.1).

We calculated the mean decrease in the total number of medication errors that cause moderate-severe patient harm (i.e. preventable adverse drug events; pADEs) with the user-tested IMG (the primary outcome of our previous clinical study [12]). We also calculated the mean decrease in the total number of pADEs with the user-tested IMG. These were calculated by extrapolating the frequency of medication errors observed with the current and user-tested IMG in our previous clinical study through our model to the total number of doses of voriconazole given in all 121 NHS organisations within our perspective (Sects 2.5 and 2.6).

We calculated the incremental cost-saving (ICS) as the difference between health service costs with the current IMG and health service costs with the user-tested IMG. This included the costs associated with medicines administration (nursing time), medication errors (correcting an error detected before administration, and treating the adverse effects of an error including extra tests, treatments and hospital length of stay), and the user-testing intervention. Due to the complexity of including insured costs, we did not consider litigation costs as they represent only 0.5% of the total cost of NHS medication errors [16].

We assumed that quality of life would be decreased by a medication error, so we assessed the benefits of user-testing using QALY decrements following a medication error. User-tested guidelines would therefore be more beneficial than current guidelines if they result in a smaller QALY decrement. We evaluated cost-effectiveness by calculating net monetary benefit (NMB). NMB represents the additional value in monetary terms generated by user-testing, so a positive NMB would indicate that user-testing is optimal. The NMB was calculated as the difference between expected incremental benefits (QALY decrements valued at a willingness-to-pay threshold of £20,000 per QALY) and expected incremental costs. We assessed uncertainty in the user-testing decision by reporting 95% credible intervals and the probability of a positive ICS or NMB, as well as cost-effectiveness acceptability curves.

### Time Horizon and Discounting

We used a 5-year time horizon. As user-testing results in changes to the design of the IMG, its effects persist and further user-testing is not required unless there are changes in practice or training. We considered 1- and 10-year time horizons in sensitivity analyses. We assumed that user-testing took place once at the start of the time horizon, so user-testing costs were not discounted. An annual discount rate of 3.5% was applied to other costs and QALYs [23].

### Model Structure

**Fig. 1:**
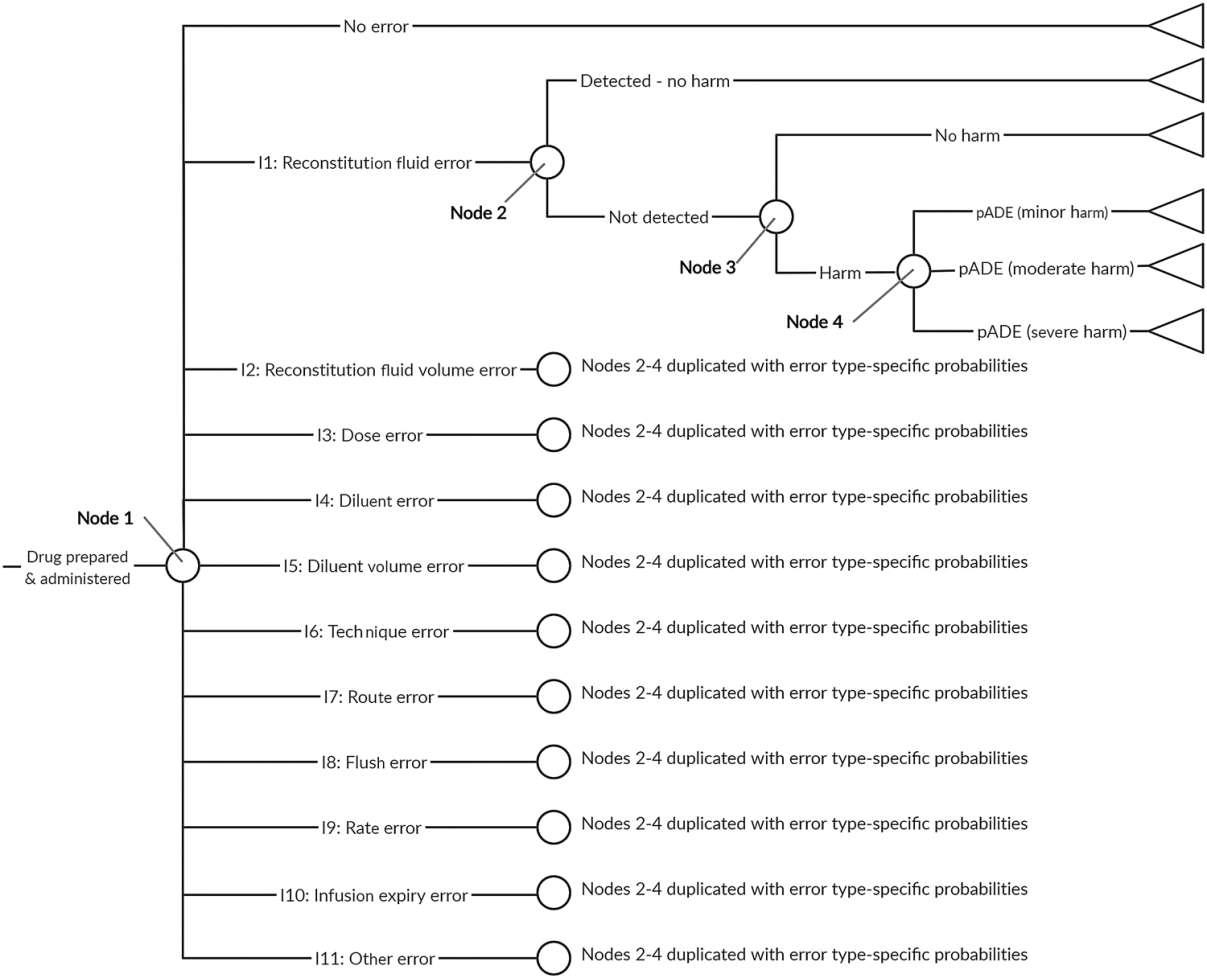
Medication errors model structure. The 11 types of medication error were the IMG-related error types used in our previous clinical study (Table A of the Online Supplemental Material) [12]. Two versions of this decision tree were populated with different probabilities at nodes 1 and 4, to describe the use of current guidelines (not user-tested) and user-tested guidelines. *IMG* injectable medicines guide, *pADE* preventable adverse drug event

The Karnon et al. decision tree model [21] considers six types of decision node in the following order: presence or absence of a prescribing error; a dispensing error or an administration error; the error type (wrong drug, dose, route or frequency); detection before administration; resultant level of harm. The present study relates only to medication administration, and we assumed that costs and QALYs associated with prescribing and dispensing are the same with user-tested and current guidelines. Therefore, we adapted the Karnon model by removing the prescribing and dispensing error nodes. The four error types in the Karnon model do not describe the range of errors observed in our clinical study, so we replaced them with the 11 types of IMG-related error from our previous study (Table A of the Online Supplemental Material) [12]. The resultant decision model used for the present study is shown in Fig. 1.

### Model Inputs to Estimate the Number of Medication Errors and Preventable Adverse Drug Events (pADEs)

Data sources used to populate the model to estimate the number of medication errors and pADEs are described in the following sections. Input parameter values and distributions for probabilistic analysis are presented in Table 2. We populated two versions of the model with different probabilities for each type of error and the level of harm from an undetected error (nodes 1 and 4 in Fig. 1) for current guidelines and user-tested guidelines.

**Table 2:** Summary of base-case model inputs

Variable	Estimates (95% confidence interval)	Distribution	Source
Probabilities of error			
Error type probabilities with current guidelines (node 1, Fig. 1)	See Table C of Online Supplemental Material	*Dirichlet (no error then error types 1 to 11):* Dirichlet (26.3, 0.3, 0.3, 10.8, 0.3, 6.3, 27.3, 0.3, 3.3, 33.3, 0.3, 27.8)	Previous clinical study [12]. *n* = 133
Error type probabilities with user-tested guidelines (node 1, Fig. 1)	See Table C of Online Supplemental Material	*Dirichlet (no error then error types 1 to 11):* Dirichlet (67.3, 0.3, 0.3, 4.3, 1.3, 0.3, 48.3, 0.3, 6.3, 11.3, 0.3, 3.3)	Previous clinical study [12]. *n* = 140
Probability an error is undetected prior to administration (node 2, Fig. 1)	0.775 (0.718–0.837) 95% prediction interval: 0.655–0.918	Log-normal (-0.25, 0.086)^a^	Random effects meta-analysis of all three randomised controlled trials [24–26] identified by a recent systematic review of the effectiveness of nurse double-checking of medicines administration [27]. The input distribution was based on the 95% prediction interval. See Online Supplemental Material page 13. *n* = 89,006
Probability an undetected error causes no harm (node 3, Fig. 1)	0.904 (0.867–0.942)	Beta (217, 23)	Proportion of observed errors from the ECLIPSE study rated as causing no harm (NCCMERP severity rating C) [8]. *n* = 240
Probabilities of minor, moderate or severe harm for a harmful, undetected error (node 4, Fig. 1)	See Table D of Online Supplemental Material	See Table 3	Previous clinical study [12]
Number of doses of intravenous voriconazole administered using IMG per annum	4000	Deterministic	Voriconazole administration and supply data provided by NHS organisations within our perspective - see Online Supplemental Material page 20
Medicine administration time			
Medicine administration time (current guidelines), min	13.3 (12.5–14.2)	Normal (13.3, 0.41)^a^	Previous clinical study [12]. *n* = 133. Converted to a cost by multiplying by the hourly cost of a band 5 nurse (see below)
Medicine administration time (user-tested guidelines), min	11.9 (11.3–12.5)	Normal (11.9, 0.31)^a^	Previous clinical study [12]. *n* = 140. Converted to a cost by multiplying by the hourly cost of a band 5 nurse (see below)
Medication error costs and QALY decrements			
Cost of an error detected before administration	£0.27 (0.03–2.39)	Log-normal (−1.29, 1.10)^b^	Karnon et al. [21] (see Online Supplemental Material pages 21–22)
Treatment cost for a minor pADE	£124 (94–163)	Log-normal (4.82, 0.14)^b^	
Treatment cost for a moderate pADE	£1252 (1092–1436)	Log-normal (7.13, 0.07)^b^	
Treatment cost for a severe pADE	£1846 (1643–2074)	Log-normal (7.52, 0.06)^b^	
QALY decrement following a minor pADE	0.004 (0.002–0.006)	Normal (0.004, 0.0011)^b^	
QALY decrement following a moderate pADE	0.035 (0.017–0.052)	Normal (0.035, 0.0089)^b^	
QALY decrement following a severe pADE	3.50 (1.87–5.13)	Normal (3.50, 0.83)^b^	
User-testing resources			
Length of pilot user-testing interview, min^c^	24 (19–28)	Normal (24, 2.2)^a^	User-testing study [11]. Converted to a cost by multiplying by sum of the hourly costs (see below) of a band 8a pharmacist (user-tester) and a band 5 nurse (participant)
Length of round 1 user-testing interview, min^c^	29 (24–33)	Normal (29, 2.5)^a^	
Length of round 2 user-testing interview, min^c^	23 (18–27)	Normal (23, 2.1)^a^	
Length of round 3 user-testing interview, min^c^	20 (17–22)	Normal (20, 1.2)^a^	
Number of pilot interviews	4	Deterministic	User-testing study [11]
Number of interviews in rounds 1–3	10	Deterministic	
Interview transcription costs, £/spoken min	1.75	Deterministic	
Length of pilot interview analysis, min^c^	94 (82–106)	Normal (94, 6.1)^a^	User-testing study [11]. Converted to a cost by multiplying by hourly cost of a band 8a pharmacist (see below)
Length of round 1 interview analysis, min^c^	76 (69–82)	Normal (76, 3.5)^a^	
Length of round 2 interview analysis, min^c^	53 (39–66)	Normal (53, 6.9)^a^	
Length of round 3 interview analysis, min^c^	50 (46–54)	Normal (50, 2.2)^a^	
Time to revise guides after round 1, min^c,d^	265 (226–304)	Normal (265, 20)^a^	User-testing study [11]. Converted to a cost by multiplying by hourly cost of a band 8a pharmacist (see below)
Time to revise guides after round 2, min^c,d^	140 (118–162)	Normal (140, 11)^a^	
Time to revise guides after round 3, min^c,d^	140 (118–162)	Normal (140, 11)^a^	
User-tester training costs (course cost, trainee time and travel), £^e^	562	Deterministic	User-testing study [11]
Equipment costs, £^e^	4	Deterministic	User-testing study [11]
Staff costs			
Hourly NHS cost of a hospital pharmacist to carry out user-testing, £	65	Deterministic	Personal and Social Services Research Unit data [28]
Hourly NHS cost of a hospital nurse as user-testing participant or to administer voriconazole, £	37	Deterministic	Personal and Social Services Research Unit data [28]
Discount rate			
Discount rate	− 0.035	Deterministic	NICE methods of technology appraisal [23].

*IMG* Injectable Medicines Guide, *NCCMERP* National Coordinating Centre of Medication Error Reporting and Prevention, *NHS* National Health Service, *NICE* National Institute for Health and Care Excellence, *QALY* quality-adjusted life year, *QoL* quality of life

^a^Distribution uses standard error

^b^Distribution uses standard deviation

^c^Previous user-testing study developed IMG guidelines for two medicines, so times halved

^d^Assumed relative standard error of 8%, (the mean relative standard error for interview and analysis time)

^e^Cost assumed to be shared between ten user tests

#### Error Type and Level of Harm Caused by a pADE

We used the frequency of errors observed in our previous clinical study (section 1.0) [12] to calculate the probabilities of each of the error types or no medication error (node 1, Fig. 1), and for the level of harm caused by a pADE resulting from each error type (node 4, Fig. 1), for both types of guideline. In our previous study, multiple errors were observed in some doses. In line with our previous analysis, in our base case we only considered the error type with the highest potential severity in each observation (Online Supplemental Material, page 3), which we used to define Dirichlet distributions for the error type probabilities (Table 2, applied to node 1 of Fig. 1) and for the probabilities of minor, moderate or severe harm related to each error type (Table 3, applied to node 4 of Fig. 1), for both the current and user-tested guidelines. In the previous clinical study, there were only 273 participants and so rare events (e.g. occurring in fewer than one in 500 doses) were unlikely to have been observed. Using the data directly would have yielded zero probabilities for such events. We therefore assumed a Dirichlet prior distribution with a count of 0.1 for all error types and severities, and used Bayesian updating with the 273-participant dataset to generate a posterior distribution. This posterior distribution would be guaranteed to have a very low, but non-zero, probability for events that did not take place in the sample.

**Table 3:** Base-case distributions for the probabilities of minor, moderate or severe harm for a harmful, undetected error for all error types and with both current and user-tested guidelines

Error type	Dirichlet distributions (minor harm, moderate harm, severe harm)	
	Current guidelines	User-tested guidelines
I1	Dirichlet (0.1, 0.1, 0.1)^a^	Dirichlet (0.1, 0.1, 0.1)^a^
I2	Dirichlet (0.1, 0.1, 0.1)^a^	Dirichlet (0.1, 0.1, 0.1)^a^
I3	Dirichlet (5.1, 5.6, 0.1)	Dirichlet (2.1, 2.1, 0.1)
I4	Dirichlet (0.1, 0.1, 0.1)^a^	Dirichlet (0.1, 1.1, 0.1)^a^
I5	Dirichlet (0.1, 6.1, 0.1)	Dirichlet (0.1, 0.1, 0.1)^a^
I6	Dirichlet (0.1, 27.1, 0.1)	Dirichlet (3.1, 45.1, 0.1)
I7	Dirichlet (0.1, 0.1, 0.1)^a^	Dirichlet (0.1, 0.1, 0.1)^a^
I8	Dirichlet (0.1, 3.1, 0.1)	Dirichlet (0.1, 6.1, 0.1)
I9	Dirichlet (4.1, 29.1, 0.1)	Dirichlet (0.1, 11.1, 0.1)
I10	Dirichlet (0.1, 0.1, 0.1)	Dirichlet (0.1, 0.1, 0.1)^a^
I11	Dirichlet (19.1, 3.6, 5.1)	Dirichlet (0.1, 2.1, 1.1)

These distributions were applied to node 4 of Fig. 1

^a^Distribution based entirely on a priori assumption of 0.1 for each error type and severity (see Sect. 2.6.1)

We also conducted a structural sensitivity analysis considering the effects of multiple errors per dose, assuming that their costs and QALY decrements were additive (Online Supplemental Material, pages 6–9).

In the clinical study, 66% of doses contained an error related to the IMG [12], whereas systematic reviews suggest that 35–48% of actual intravenous doses contain an error [3, 4]. Therefore, another sensitivity analysis explored the effects of adjusting the medication error frequency with current guidelines to a rate similar to that reported in practice (32%) while maintaining an unchanged relative reduction in error frequency after user-testing, by adjusting the probabilities associated with nodes 1 and 4 of Fig. 1 (see Tables I–L of the Online Supplemental Material). To explore the consequences of user-testing being less effective when medication errors are less common (i.e. medicines that are simpler to prepare and administer), a further sensitivity analysis considered the relative effects of user-testing being halved when the frequency of medication errors is reduced to 32% of doses (Tables I–L of the Online Supplemental Material).

#### Probability of Error Detection

The Karnon model includes a step accounting for the possible detection of an error before it reaches the patient (node 2, Fig. 1). In this study, the only opportunity to detect an error is if a prepared dose is double-checked by a second nurse, which is recommended practice within the NHS [29]. We therefore used the risk ratio for a medication administration error following a double-check by a nurse (compared with no double-check) to populate the model (node 2 of Fig. 1). This combines the probability of a double check being completed and the probability of it detecting an error. A random effects meta-analysis of published data (Online Supplemental Material, page 13) yielded the parameters described in Table 2. The Online Supplemental Material (page 13) describes sensitivity analyses of this risk ratio.

#### Probability that an Undetected Error Causes No Harm

There are few data on the actual consequences of medication errors, as research tends to report the potential consequences had an error not been intercepted. However, not every medication error that reaches a patient produces its potential harm [16, 22]. We specified that for our model this probability should relate to UK intravenous medicines administration, due to the high risk of this route of administration and significant international differences between medication administration systems. A recent systematic review of intravenous medication errors in the UK identified no suitable data [30]. However, a more recent UK study identified intravenous infusion errors using a point prevalence approach, in which errors were identified after administration had commenced [8]. A total of 240 errors were observed, of which 217 were rated as causing no harm [31]. We therefore populated node 3 of Fig. 1 with the parameters described in Table 2. As these data are derived from only one study, we included both increased (0.99) and decreased (0.75) probabilities in our sensitivity analyses (Table M of the Online Supplemental Material).

#### Number of Doses of Voriconazole Administered Using the Injectable Medicines Guide (IMG) per Annum

We used the number of doses of voriconazole administered using the IMG per annum to convert the probability of each outcome of the decision tree (Fig. 1) into an estimated number of patients. Using voriconazole administration and supply data provided by NHS organisations within our perspective (Online Supplemental Material, page 20), we estimated this value to be between 4130 and 22,980 doses per annum. We used a conservative estimate of 4000 doses in our base-case analysis and carried out a sensitivity analysis with 20,000 doses.

### Model Inputs to Estimate Costs and Benefits

We converted the estimated number of medication errors and pADEs into estimated health service costs (see Sect. 2.3) and QALY decrements for a pADE, using the parameters described in Table 2.

#### Nurse Medicine Administration Time and Costs

Our clinical study measured the time taken to prepare and administer a dose of voriconazole with current and user-tested guidelines [12]. We used these data to define normal distributions in our probabilistic analyses (Table 2).

#### Medication Error Costs and Quality-Adjusted Life Year (QALY) Decrements

Recent reviews found very few published estimates of the UK cost of a medication error [15, 16]. The cost estimates synthesised by one of these reviews [13] were not suitable for the present study, because they do not describe costs for different levels of pADE harm. Therefore, they would not allow use of the complete medication error frequency data available from our clinical study, which give an estimate of pADE harm that is specific to the context of the present investigation. Of the available estimates, the most appropriate for this study were those published by Karnon et al*.* [19], as they specifically relate to errors involving medicines administration in hospital, without restriction to specific types of medicines. In addition, Karnon’s data describe costs for three levels of pADE harm, as required for our model (Fig. 1). They include the costs of correcting an error detected before administration and treating the adverse effects of a pADE, including extra tests, treatments and hospital length of stay. Karnon’s data also include hypothetical QALY decrement ranges following a pADE based on limited data and research team discussion [21], which is fully described in their final report [32]. As this approach was not based on primary data, its validity is limited, but these data are the only available estimates. The Online Supplemental Material (page 21) describes how we used these data (uprated to 2018 values using the Hospital and Community Health Services Index [28]) to derive the model inputs for medication error costs and QALY decrements (Table 2). Given high levels of uncertainty about these parameters, additional sensitivity analyses considered scenarios where they were both higher and lower (Tables P and Q of the Online Supplemental Material). We assumed that other costs associated with preparing and administering a dose of voriconazole were identical to both guidelines and did not include them.

#### User-Testing Costs

We calculated the NHS cost of user-testing using resource use data from our previous study (Table 2) [11]. This assumed an approach to user-testing similar to that currently used to write the IMG: user-testing shared between organisations within our perspective and performed by a hospital pharmacist with hospital nurses as interview participants. We assumed that other costs associated with preparing and delivering the IMG were identical to both guidelines and did not include them.

### Model Analysis and Validation

We developed the model in Excel, using Monte Carlo simulation with 20,000 samples to propagate the joint uncertainty in model inputs into the outcomes, as this was found sufficient for graphical convergence of medication errors, costs and NMB. We reported costs in pounds (£) for the 2018 price year.

We established face validity by discussion with an advisory group (doctors, nurses, pharmacists and IMG advisory board members) and within the research team (health-economic modellers, medication-safety researchers and pharmacists). We systematically checked internal validity by assessing core calculations and applying a series of logical checks to ensure the direction of predictions was consistent. External and cross validity were more difficult to establish, as there are few comparable studies.

## Results

**Fig. 2:**
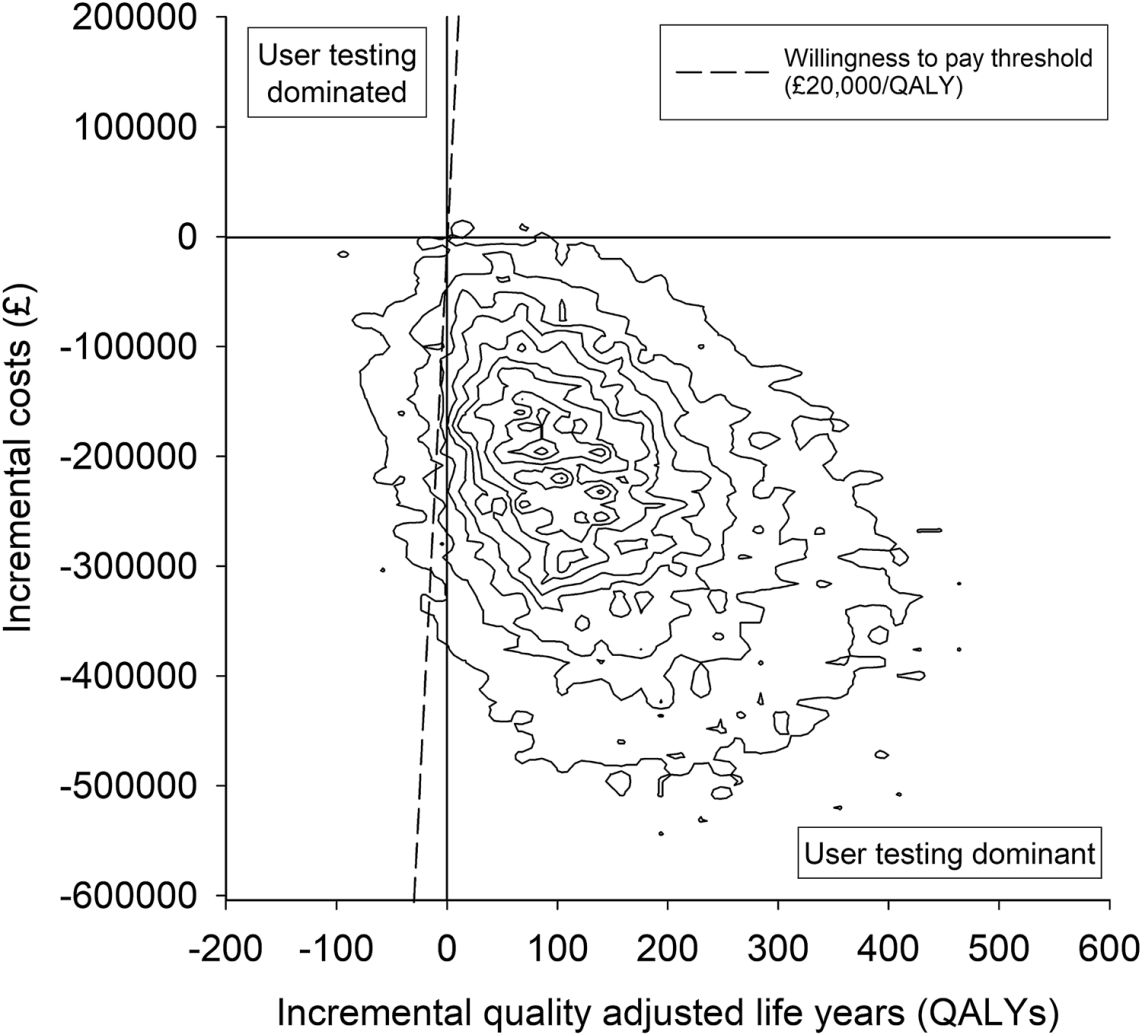
Cost-effectiveness plane of the base-case probabilistic sensitivity analysis. Each contour represents 10% of the simulation results. A positive incremental cost saving in Tables 4 and 5 is equivalent to a negative incremental cost in this figure

**Fig. 3:**
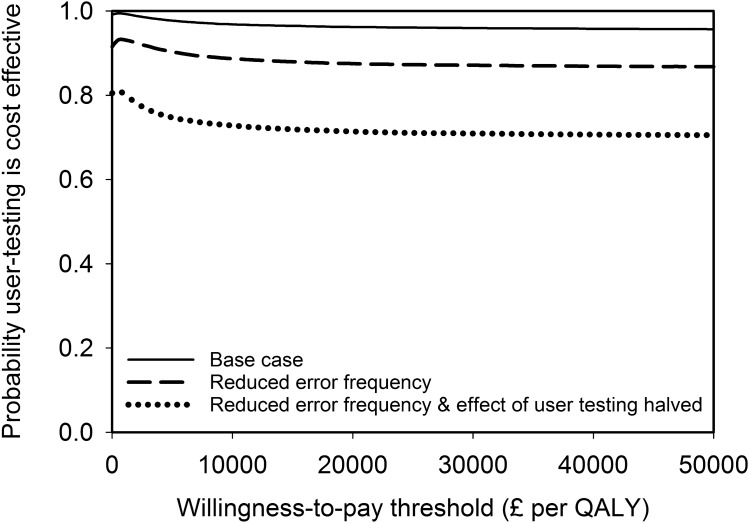
Cost-effectiveness acceptability curve for the base-case and two sensitivity analyses. *QALY* quality-adjusted life year

The results from the base-case analysis for the whole cohort of patients within an NHS payer perspective are presented in Table 4. There were 157 fewer moderate-severe pADEs when user-tested guidelines were used, but with considerable uncertainty about this estimate. There was greater certainty around the reduction of 411 in the total number of pADEs when user-tested guidelines were used. There were fewer QALY decrements associated with the use of the user-tested guidelines and ICS favoured user-testing, with a 99% chance of being cost-saving. The cost-effectiveness plane (Fig. 2) indicates that 94.7% of simulations were clustered in the quadrant where user-testing was dominant. At a willingness-to-pay threshold of £20,000 per QALY, the NMB was over £3 million but with considerable uncertainty and a 96% chance of user-testing being cost-effective. The cost-effectiveness acceptability curve shows that this high likelihood of cost-effectiveness is maintained over a wide range of willingness-to-pay thresholds (Fig. 3).

**Table 4:** Base-case analysis costs and outcomes of using current and user-tested guidelines to support the administration of intravenous voriconazole for the entire cohort of patients over a time horizon of 5 years

	Mean with current guidelines (95% credible interval)	Mean with user-tested guidelines (95% credible interval)	Mean difference (95% credible interval)	Probability user-tested guidelines superior^a^
Number of moderate-severe pADEs	885 (542–1330)	728 (441–1102)	157 (−13 to 363)	0.96
Total number of pADEs	1202 (748–1775)	792 (481–1191)	411 (210 to 675)	1.00
QALY decrements	238.8 (83.9–513.5)	91.4 (26.0–237.6)	147.5 (−24.9 to 406.1)	0.95
Total user-testing cost, £	–	6317 (6012–6627)	–	–
Health system costs, £	1,225,800 (799,808–1,779,547)	978,540 (635,010–1,434,142)	Incremental cost-saving: 240,943 (43,527 to 491,576)	0.99
Net monetary benefit, £^b^	–	–	3,190,064 (−346,709 to 8,480,665)	0.96

*pADE* preventable adverse drug effect, *QALY* quality-adjusted life year

^a^Superiority corresponds to lower pADEs, costs, or QALY decrements

^b^Willingness-to-pay threshold of £20,000 per QALY

**Table 5:** Selected sensitivity analysis costs and outcomes of using current and user-tested guidelines to support the administration of intravenous voriconazole for the entire cohort of patients over a time horizon of 5 years. Table M of the Online Supplemental Material shows the results of all sensitivity analyses

Parameter varied from base-case analysis^a^	Mean decrease with user-testing (95% credible interval)			Incremental cost-saving		Net monetary benefit^b^	
	Moderate-severe pADEs	All pADEs	QALY decrements	Mean (95% credible interval), £	Probability user-testing cost-saving	Mean (95% credible interval), £	Probability user-testing cost-effective
Base case (for comparison)	157 (−13 to 363)	411 (210–675)	147.5 (−24.9 to 406.1)	240,943 (43,527–491,576)	0.99	3,190,064 (−346,709 to 8,480,665)	0.96
Time horizon reduced to 1 year	31 (−3 to 73)	82 (42–136)	31.2 (−5.2 to 87.1)	46,648 (3,177–101,006)	0.98	669,944 (−74,287 to 1,815,983)	0.96
Time horizon increased to 10 years	315 (−32 to 728)	823 (420–1,356)	265.8 (−46.5 to 742.3)	449,553 (79,168–911,049)	0.99	5,766,532 (−612,727 to 15,470,264)	0.96
Number of doses of intravenous voriconazole administered using IMG per annum increased to 20,000	787 (−80 to 1,822)	2,057 (1,039–3,402)	730.5 (−133.4 to 2,041.3)	1,231,112 (224,306–2,508,969)	0.99	15,840,983 (−1,816,590 to 42,701,213)	0.96
Undetected error frequency with current guidelines reduced to 32%, with unchanged relative reduction in error frequency after user-testing	80 (−77 to 252)	208 (36–421)	75.4 (−64.7 to 269.0)	127,725 (−57,148–335,690)	0.92	1,636,052 (−1,253,565 to 5,611,961)	0.88
Undetected error frequency with current guidelines reduced to 32% and relative effect of user-testing halved	41 (−110 to 223)	119 (−49–316)	40.6 (−125.9 to 237.5)	81,539 (−106,273–285,048)	0.81	892,882 (−2,526,604 to 4,916,336)	0.71

^a^See Tables I to L of the Online Supplemental Material

^b^Willingness-to-pay threshold = £20,000 per QALY

*pADE* preventable adverse drug event, *QALY* quality-adjusted life year

Table 5 shows the results of selected sensitivity analyses (shown in full in Table M of the Online Supplemental Material). Uncertainty about the time horizon (Table 5), probability of detecting an error, probability of an error causing no harm, annual number of doses of intravenous voriconazole (Table 5), QALY decrement distributions and medication error cost distributions changes the magnitude of the outcomes but does not alter optimal strategy. Likewise, the magnitude of outcomes changes but the optimal strategy does not alter when all errors (rather than just the most severe error for each dose) are considered, or when the original NMB calculations used by Karnon et al. are applied. However, when the frequency of medication errors with current guidelines was reduced from 62% of doses (base case) to 32% of doses (similar to the frequency described by systematic reviews [3, 4]), the probability that user-testing is cost-effective is reduced and there is greater uncertainty about the ICS and NMB (Table 5). This reduction is even greater if the effectiveness of user-testing in reducing medication errors is also halved. The cost-effectiveness acceptability curves for these last two sensitivity analyses are shown in Fig. 3.

## Discussion

This paper reports the development of a model to assess the cost-effectiveness of user-testing injectable medicines guidelines for use by nurses preparing intravenous medicines. The results suggest that for high-risk situations (e.g. an unfamiliar drug), user-testing reduces the number of pADEs and is both cost-saving and cost-effective. These findings were robust to many sources of uncertainty. For lower-risk situations, there is greater uncertainty about these outcomes, especially if user-testing is relatively less effective, although there is still > 70% probability that user-testing is cost-effective. Although these findings are specific to the UK, they are likely to be applicable in any setting that uses similar intravenous medicines administration systems, as user-testing is a relatively inexpensive intervention and a reduction in pADEs is likely to result in large cost reductions.

There are no other published economic evaluations of user-testing for improving medicines safety. The cost-effectiveness of several other hospital medicines safety interventions has been assessed. We based the model described in this study on one developed by Karnon et al., who used this approach to evaluate the cost-effectiveness of three medicines safety interventions: computerised physician order entry (CPOE), additional ward pharmacists, and bar coding of medicines [21]. In contrast to the present study, it was uncertain whether any of these interventions were cost-saving, but all three were strongly cost-effective in terms of NMB. Karnon et al. later adapted this model to examine the cost-effectiveness of five medicines reconciliation interventions during hospital admission [33]. As in the present study, three interventions were found to be cost-saving while also increasing QALYs. A study using a different model concluded that the use of clinical pharmacists to detect errors in prescriptions for antineoplastic drugs was cost-effective, with estimated annual savings of €249,844 for one hospital [18]. Unlike the present study, other economic evaluations of hospital medicines safety projects have only considered intervention costs and not the costs avoided by reducing pADEs. For example, Vermeulen et al. estimated an incremental cost-effectiveness ratio (ICER) of €322.70 per pADE avoided by a CPOE system [17]. Based on the mean user-testing cost and mean total number of pADEs avoided (Table 4), the equivalent ICER for this study is £15.41 per pADE avoided.

These comparisons emphasise that user-testing is a relatively low-cost patient safety intervention, with a small initial cost and little requirement for repetition or maintenance (unlike training or CPOE for example). The present study assumed that user-testing was carried out by NHS-employed pharmacists. However, utilising the expertise of user-testing specialists, with the input of specialists in information writing and design, may produce even more effective guidelines. The time to complete the process might also be reduced, but with some increased costs. In addition, the present study describes an ‘intensive’ user-testing process, where the guideline for one medicine was tested with 30 nurses. However, some of the findings from this process are applicable to the IMG guidelines for all other medicines. Such learning will reduce the overall amount of testing required to improve the guidelines for all 350 medicines in the IMG, thus further improving the cost-effectiveness of user-testing.

### Limitations

One limitation of our study is that the modelled results draw on an in situ simulation rather than the investigation of actual patient care [12]. This may have changed participants’ practice. Our previous simulation involved an unfamiliar medicine, whereas in day-to-day practice nurses are familiar with most of the medicines they administer. While the administration of lower-risk medicines was explored in the sensitivity analysis, it is not certain if these analyses were reasonable and explored the full range of possibilities. In addition, the classification of the potential level of harm caused by each error was based on the subjective judgement of an expert panel, although there was high agreement among this panel (Cronbach’s alpha 0.93), and it correctly assigned the level of harm for 13 of 15 medication errors with a known outcome [12]. A further limitation is the limited amount of empirical data used by Karnon et al. to derive their QALY decrements following a pADE and the lack of subsequent validation of these parameters [21], although sensitivity analyses found that the cost-effectiveness of user-testing was robust to large changes in these QALY decrements. Finally, a broader societal perspective would have included a greater range of costs, such as social care following permanent injury by a medication error.

### Implications

These results suggest that the IMG should adopt the process of user-testing to produce guidelines for high-risk medicines. Evaluation of the impact of this change could inform a similar decision in relation to lower-risk medicines. As user-testing is a low-cost intervention and the costs of pADEs are relatively much higher, this approach should also be considered for other types of written guidelines for health professionals, including those both related and unrelated to medicines. Economic evaluation is an important tool to support the introduction of medicines safety interventions but is currently limited by lack of consensus and rigour in relation to how the costs and consequences of medication errors should be calculated [34]. Future research should aim to determine the most appropriate methods for calculating medication error costs and consequences in different contexts.

## Conclusion

There is a high probability that user-testing injectable medicines guidelines for nurses preparing intravenous medicines in clinical areas is both cost-effective (96%) and cost-saving (99%) for high-risk medicines. The probability that user-testing is cost-effective and cost-saving is reduced for lower-risk medicines, but is still 71% and 80% (respectively), as user-testing is a low-cost intervention.

## Supplementary Information

Below is the link to the electronic supplementary material.

Supplementary file1 (PDF 421 kb)

## Declarations

### Availability of Data and Material

The model and supporting data created during this research are openly available from the University of Bath Research Data Archive at https://doi.org/10.15125/BATH-00960.

### Code Availability

The model created during this research is openly available from the University of Bath Research Data Archive at https://doi.org/10.15125/BATH-00960.
