# Supplementary material for: Costs and Cost-Effectiveness of User-Testing of Health Professionals’ Guidelines to Reduce the Frequency of Intravenous Medicines Administration Errors by Nurses in the United Kingdom: A Probabilistic Model Based on Voriconazole Administration
Source: Appl Health Econ Health Policy. Author manuscript; Available in PMC 2022 Jan 17. (PMC8752547; doi:10.1007/s40258-021-00675-z)
Supplement: Online Supplementary Material [file EMS134947-supplement-Online_Supplementary_Material.pdf]

Applied Health Economics and Health Policy

Costs and cost-effectiveness of user-testing of health professionals' guidelines to reduce the frequency of intravenous medicines administration errors by nurses in the United Kingdom: a probabilistic model based on voriconazole administration

Running title: Cost-effectiveness of user-testing guidelines

Matthew D. Jones (ORCID 0000-0002-2617-4098)[1*], Bryony Dean Franklin (ORCID 0000-0002-2892-1245)[2,3], DK Raynor (ORCID 0000-0003-0306-5275)[4,5], Howard Thom (0000-0001-8576-5552)[6], Margaret C Watson (0000-0002-8198-9273)[1,7] and Rebecca Kandiyali (ORCID 0000-0001-8566-9536)[6].

[1]Department of Pharmacy and Pharmacology, University of Bath, Bath, UK.

[2]UCL School of Pharmacy, London, UK.

[3]Centre for Medication Safety and Service Quality, Pharmacy Department, Imperial College Healthcare NHS Trust, London, UK

[4]Luto Research, Leeds, UK.

[5]School of Healthcare, University of Leeds, Leeds, UK.

[6]Bristol Medical School, University of Bristol, Bristol, UK.

[7]Strathclyde Institute of Pharmacy and Biomedical Sciences, University of Strathclyde, Glasgow, UK.

*Corresponding author:

E-mail: M.D.Jones@bath.ac.uk

Table A: Types of IMG-related error used in our previous clinical study [1]. An *IMG-related error was defined as an error in a process that required use of information from the IMG.*

**Error code**	**Error type description**
I1	Wrong reconstituting fluid
I2	Wrong reconstituting fluid volume
I3	Dose discrepancy
I4	Wrong diluent
I5	Wrong diluent volume
I6	Incorrect technique (IMG-related)
I7	Wrong route
I8	Flush error
I9	Rate discrepancy
I10	Infusion expiry error
I11	Other IMG related error

IMG = Injectable Medicines Guide

Number of doses in our clinical study for which each IMG-related error type had the highest potential severity

We determined the number of doses in our clinical study for which each IMG-related error type had the highest potential severity, which were already stratified by potential severity category (minor, moderate or major) by an expert panel [1]. Where two error types with an equal highest potential severity score were observed for one dose, 0.5 was added to the total of each of the relevant error types. Table B of Online Resource (below) shows these data.

Table B: the number of doses for which each Injectable Medicines Guide-related error type had the highest potential severity in our previous study [1]. Data are stratified according to the potential severity category of the error (determined by an expert panel).

	**Potential severity of error**	**Error type (see Table A for description)**											
		**No error**	**I1**	**I2**	**I3**	**I4**	**I5**	**I6**	**I7**	**I8**	**I9**	**I10**	**I11**
**Current guidelines (n = 133)**	Minor	-	0	0	5	0	0	0	0	0	4	0	19
	Moderate	-	0	0	5.5[a]	0	6	27	0	3	29[a]	0	3.5[a]
	Major	-	0	0	0	0	0	0	0	0	0	0	5
	Total	26	0	0	11	0	6	27	0	3	34	0	28
**User-tested guidelines (n = 140)**	Minor	-	0	0	2	0	0	3	0	0	0	0	0
	Moderate	-	0	0	2	1	0	45	0	6	11	0	2
	Major	-	0	0	0	0	0	0	0	0	0	0	1
	Total	67	0	0	4	1	0	48	0	6	11	0	3

[a]In one case, a dose error (I3) and a rate error (I9) had the equal highest potential severity score. For another case, a rate error (I9) and an other error (I11) had the equal highest potential severity score. In these cases, 0.5 was added to each of the relevant error types.

Table C: Base case estimates of error type probabilities with both current and user-tested guidelines (node 1, Figure 1)

	**Error type (see Table A for description)**											
	**No error**	**I1**	**I2**	**I3**	**I4**	**I5**	**I6**	**I7**	**I8**	**I9**	**I10**	**I11**
**Current guidelines**	0.193	0.002	0.002	0.079	0.002	0.046	0.200	0.002	0.024	0.244	0.002	0.204
**User-tested guidelines**	0.469	0.002	0.002	0.030	0.009	0.002	0.336	0.002	0.044	0.079	0.002	0.023

Table D: Base case estimates of the probabilities of minor, moderate or severe harm for a harmful, undetected error for all error types and with both current and user-tested guidelines (node 4, Figure 1)

		**Error type (see Table A for description)**										
		**I1**	**I2**	**I3**	**I4**	**I5**	**I6**	**I7**	**I8**	**I9**	**I10**	**I11**
**Current guidelines**	**Minor harm**	0.333	0.333	0.472	0.333	0.016	0.004	0.333	0.030	0.123	0.333	0.687
	**Moderate harm**	0.333	0.333	0.519	0.333	0.968	0.993	0.333	0.939	0.874	0.333	0.129
	**Major harm**	0.333	0.333	0.009	0.333	0.016	0.004	0.333	0.030	0.003	0.333	0.183
**User-tested guidelines**	**Minor harm**	0.333	0.333	0.488	0.077	0.333	0.064	0.333	0.016	0.009	0.333	0.030
	**Moderate harm**	0.333	0.333	0.488	0.846	0.333	0.934	0.333	0.968	0.982	0.333	0.636
	**Major harm**	0.333	0.333	0.023	0.077	0.333	0.002	0.333	0.016	0.009	0.333	0.333

Structural sensitivity analysis considering the effects of multiple errors per dose

Our structural sensitivity analysis of the effects of multiple errors per dose was based on the total number of errors reported in our clinical study (Table E of Online Resource) [1] and assumed that their costs and QALY decrements were additive (Tables F-H of the Online Resource). We chose to use this scenario as a sensitivity analysis (rather than the base case), as costs and QALY decrements are unlikely to be fully additive. For example, increased hospital length of stay or laboratory tests to treat one pADE may also treat a second simultaneous pADE. We therefore decided to use the more conservative 'single highest potential severity error' approach as the base case.

Table E: Total number of injectable medicine guide-related errors observed during our previous clinical study [1], categorised by potential severity and error type. Table reproduced under CC BY license: https://creativecommons.org/licenses/by/4.0/.

Error code	Error type	Current guidelines (control) (n=133 simulations)				User-tested guidelines (n=140 simulations)			
		Minor	Moderate	Severe	Total	Minor	Moderate	Severe	Total
**I1**	**Wrong reconstituting fluid**	0	0	0	0	0	0	0	0
**I2**	**Wrong reconstituting fluid volume**	1	0	0	1	0	0	0	0
**I3**	**Dose discrepancy**	13	16	0	29	4	6	0	10
**I4**	**Wrong diluent**	0	0	0	0	0	1	0	1
**I5**	**Wrong diluent volume**	0	6	0	6	0	0	0	0
**I6**	**Incorrect technique (IMG related)**	1	55	0	56	6	58	0	64
**I7**	**Wrong route**	0	0	0	0	0	0	0	0
**I8**	**Flush error**	0	12	0	12	0	12	0	12
**I9**	**Rate discrepancy**	10	30	0	40	1	12	0	13
**I10**	**Infusion expiry error**	0	0	0	0	0	0	0	0
**I11**	**Other IMG-related error**	23	4	5	32	1	2	1	4
	**Total**	**48**	**123**	**5**	**176**	**12**	**91**	**1**	**104**

IMG = Injectable Medicines Guide

Table F: Multiple error structural sensitivity analysis estimates of error type probabilities and distributions with both current and user-tested guidelines. In this analysis, each error type was considered separately and described using a beta distribution based on the total number of errors observed in our previous clinical study, as shown in Table B. These distributions were applied to node 1 of Figure 1.

Error type (see Table A for description)	Current guidelines			User-tested guidelines		
	Probability (95% confidence interval)	Beta distribution parameters for probabilistic analyses		Probability (95% confidence interval)	Beta distribution parameters for probabilistic analyses	
		α	β		α	β
**I1**	0.002 (0.000 to 0.010)	0.3	136.0	0.002 (0.000 to 0.010)	0.3	143.0
**I2**	0.01 (0.000 to 0.026)	1.3	135.0	0.002 (0.000 to 0.010)	0.3	143.0
**I3**	0.215 (0.146 to 0.284)	29.3	107.0	0.072 (0.030 to 0.114)	10.3	133.0
**I4**	0.002 (0.000 to 0.010)	0.3	136.0	0.009 (0.000 to 0.025)	1.3	142.0
**I5**	0.046 (0.011 to 0.081)	6.3	130.0	0.002 (0.000 to 0.010)	0.3	143.0
**I6**	0.413 (0.331 to 0.495)	56.3	80.0	0.449 (0.368 to 0.530)	64.3	79.0
**I7**	0.002 (0.000 to 0.010)	0.3	136.0	0.002 (0.000 to 0.010)	0.3	143.0
**I8**	0.09 (0.042 to 0.138)	12.3	124.0	0.086 (0.040 to 0.132)	12.3	131.0
**I9**	0.296 (0.219 to 0.372)	40.3	96.0	0.093 (0.045 to 0.140)	13.3	130.0
**I10**	0.002 (0.000 to 0.010)	0.3	136.0	0.002 (0.000 to 0.010)	0.3	143.0
**I11**	0.237 (0.166 to 0.308)	32.3	104.0	0.03 (0.002 to 0.058)	4.3	139.0

Table G: Multiple error structural sensitivity analysis estimate of the probabilities of minor, moderate or severe harm for a harmful, undetected error for all error types and with both current and user-tested guidelines

		Error type (see Table A for description)										
		**I1**	**I2**	**I3**	**I4**	**I5**	**I6**	**I7**	**I8**	**I9**	**I10**	**I11**
**Current guidelines**	**Minor harm**	0.333	0.846	0.447	0.333	0.016	0.020	0.333	0.008	0.251	0.333	0.715
	**Moderate harm**	0.333	0.077	0.549	0.333	0.968	0.979	0.333	0.984	0.747	0.333	0.127
	**Major harm**	0.333	0.077	0.003	0.333	0.016	0.002	0.333	0.008	0.002	0.333	0.158
**User-tested guidelines**	**Minor harm**	0.333	0.333	0.398	0.077	0.333	0.095	0.333	0.008	0.083	0.333	0.256
	**Moderate harm**	0.333	0.333	0.592	0.846	0.333	0.904	0.333	0.984	0.910	0.333	0.488
	**Major harm**	0.333	0.333	0.010	0.077	0.333	0.002	0.333	0.008	0.008	0.333	0.256

Table H: Multiple error structural sensitivity analysis distributions for the probabilities of minor, moderate or severe harm for a harmful, undetected error for all error types and with both current and user-tested guidelines. These distributions were applied to node 4 of Figure 1.

Error type (see Table A for description)	Dirichlet distributions (minor harm, moderate harm, severe harm)	
	**Current guidelines**	**User-tested guidelines**
**I1**	Dirichlet (0.1, 0.1, 0.1)	Dirichlet (0.1, 0.1, 0.1)
**I2**	Dirichlet (1.1, 0.1, 0.1)	Dirichlet (0.1, 0.1, 0.1)
**I3**	Dirichlet (13.1, 16.1, 0.1)	Dirichlet (4.1, 6.1, 0.1)
**I4**	Dirichlet (0.1, 0.1, 0.1)	Dirichlet (0.1, 1.1, 0.1)
**I5**	Dirichlet (0.1, 6.1, 0.1)	Dirichlet (0.1, 0.1, 0.1)
**I6**	Dirichlet (1.1, 55.1, 0.1)	Dirichlet (6.1, 58.1, 0.1)
**I7**	Dirichlet (0.1, 0.1, 0.1)	Dirichlet (0.1, 0.1, 0.1)
**I8**	Dirichlet (0.1, 12.1, 0.1)	Dirichlet (0.1, 12.1, 0.1)
**I9**	Dirichlet (10.1, 30.1, 0.1)	Dirichlet (0.1, 12.1, 0.1)
**I10**	Dirichlet (0.1, 0.1, 0.1)	Dirichlet (0.1, 0.1, 0.1)
**I11**	Dirichlet (23.1, 4.1, 5.1)	Dirichlet (1.1, 2.1, 1.1)

Table I: Reduced error frequency sensitivity analyses: estimates of error type probabilities with both current and user-tested guidelines (applied to node 1 of Figure 1)

	Error type (see Table A for description)											
	**No error**	**I1**	**I2**	**I3**	**I4**	**I5**	**I6**	**I7**	**I8**	**I9**	**I10**	**I11**
**Current guidelines with decreased frequency of medication errors (32%)**	0.584	0.002	0.002	0.041	0.002	0.024	0.101	0.002	0.013	0.123	0.002	0.103
**User-tested guidelines with decreased frequency of medication errors**	0.723	0.002	0.002	0.016	0.006	0.002	0.169	0.002	0.023	0.040	0.002	0.013
**User-tested guidelines with decreased frequency of medication errors AND relative effect of user-testing halved**	0.664	0.002	0.002	0.027	0.004	0.013	0.133	0.002	0.018	0.079	0.002	0.055

Table J: Reduced error frequency sensitivity analyses: distributions for error type probabilities with both current and user-tested guidelines (applied to node 1 of Figure 1)

	**Dirichlet distributions (no error then error types 1 to 11)**
**Current guidelines with decreased frequency of medication errors (32%)**	Dirichlet (79.8, 0.3, 0.3, 5.55, 0.3, 3.3, 13.8, 0.3, 1.8, 16.8, 0.3, 14.05)
**User-tested guidelines with decreased frequency of medication errors**	Dirichlet (103.8, 0.3, 0.3, 2.3, 0.8, 0.3, 24.3, 0.3, 3.3, 5.8, 0.3, 1.8)
**User-tested guidelines with decreased frequency of medication errors AND relative effect of user-testing halved**	Dirichlet (95.3, 0.3, 0.3, 3.925, 0.55, 1.8, 19.05, 0.3, 2.55, 11.3, 0.3, 7.925)

Table K: Reduced error frequency sensitivity analyses: estimates of the probabilities of minor, moderate or severe harm for a harmful, undetected error for all error types and with both current and user-tested guidelines (applied to node 1 of Figure 1)

		**Error type (see Table A for description)**										
		**I1**	**I2**	**I3**	**I4**	**I5**	**I6**	**I7**	**I8**	**I9**	**I10**	**I11**
**Current guidelines with decreased frequency of medication errors (32%)**	**Minor harm**	0.333	0.333	0.468	0.333	0.030	0.007	0.333	0.056	0.125	0.333	0.683
	**Moderate harm**	0.333	0.333	0.514	0.333	0.939	0.986	0.333	0.889	0.869	0.333	0.132
	**Major harm**	0.333	0.333	0.018	0.333	0.030	0.007	0.333	0.056	0.006	0.333	0.185
**User-tested guidelines with decreased frequency of medication errors**	**Minor harm**	0.333	0.333	0.478	0.125	0.333	0.066	0.333	0.030	0.017	0.333	0.056
	**Moderate harm**	0.333	0.333	0.478	0.750	0.333	0.930	0.333	0.939	0.966	0.333	0.611
	**Major harm**	0.333	0.333	0.043	0.125	0.333	0.004	0.333	0.030	0.017	0.333	0.333
**User-tested guidelines with decreased frequency of medication errors AND relative effect of user-testing halved**	**Minor harm**	0.333	0.333	0.471	0.182	0.056	0.045	0.333	0.039	0.097	0.333	0.612
	**Moderate harm**	0.333	0.333	0.503	0.636	0.889	0.950	0.333	0.922	0.894	0.333	0.186
	**Major harm**	0.333	0.333	0.025	0.182	0.056	0.005	0.333	0.039	0.009	0.333	0.202

Table L: Reduced error frequency sensitivity analyses: distributions for the probabilities of minor, moderate or severe harm for a harmful, undetected error for all error types and with both current and user-tested guidelines (applied to node 1 of Figure 1)

Error type (see Table A for description)	Dirichlet distributions (minor harm, moderate harm, severe harm)		
	Current guidelines with decreased frequency of medication errors (32%)	User-tested guidelines with decreased frequency of medication errors	User-tested guidelines with decreased frequency of medication errors AND relative effect of user-testing halved
**I1**	Dirichlet (0.1, 0.1, 0.1)	Dirichlet (0.1, 0.1, 0.1)	Dirichlet (0.1, 0.1, 0.1)
**I2**	Dirichlet (0.1, 0.1, 0.1)	Dirichlet (0.1, 0.1, 0.1)	Dirichlet (0.1, 0.1, 0.1)
**I3**	Dirichlet (2.6, 2.85, 0.1)	Dirichlet (1.1, 1.1, 0)	Dirichlet (1.85, 1.975, 0.1)
**I4**	Dirichlet (0.1, 0.1, 0.1)	Dirichlet (0.1, 0.6, 0.1)	Dirichlet (0.1, 0.35, 0.1)
**I5**	Dirichlet (0.1, 3.1, 0.1)	Dirichlet (0.1, 0.1, 0.1)	Dirichlet (0.1, 1.6, 0.1)
**I6**	Dirichlet (0.1, 13.6, 0.1)	Dirichlet (1.6, 22.6, 0.1)	Dirichlet (0.85, 18.1, 0.1)
**I7**	Dirichlet (0.1, 0.1, 0.1)	Dirichlet (0.1, 0.1, 0.1)	Dirichlet (0.1, 0.1, 0.1)
**I8**	Dirichlet (0.1, 1.6, 0.1)	Dirichlet (0.1, 3.1, 0.1)	Dirichlet (0.1, 2.35, 0.1)
**I9**	Dirichlet (2.1, 14.6, 0.1)	Dirichlet (0.1, 5.6, 0.1)	Dirichlet (1.1, 10.1, 0.1)
**I10**	Dirichlet (0.1, 0.1, 0.1)	Dirichlet (0.1, 0.1, 0.1)	Dirichlet (0.1, 0.1, 0.1)
**I11**	Dirichlet (9.6, 1.85, 2.6)	Dirichlet (0.1, 1.1, 0.6)	Dirichlet (4.85, 1.475, 1.6)

Risk ratio for a medication administration error following a double-check by a nurse (compared with no double-check)

We identified a recent systematic review of this topic [2]; literature searches did not identify subsequent primary research. The review identified three randomised controlled trials relating to 89,006 observations of different types of dose [3-5]. We used random effects meta-analysis (using STATA v16.0) to combine these results and calculate a risk ratio of 0.775 (95% confidence interval: 0.718 to 0.837, 95% prediction interval: 0.655 to 0.918, $\tau^2 = 0.00$, $I^2 = 0.00$). This risk ratio was used to populate node 2 of the model (Figure 1) using a log-normal distribution based on the prediction interval (mean = –0.25, standard error = 0.086).

However, as the quality of this best available evidence is relatively poor [2], it is not specific to intravenous medicines and double-checking policies vary between NHS hospitals [6], we also included alternative risk ratios in sensitivity analyses to simulate outcomes when double-checking is much more effective at detecting errors (risk ratio = 0.1) and in hospitals that do not have a double checking policy (risk ratio = 1.0) (Table M of Online Resource).

Table M: input parameters varied during sensitivity analyses and the resultant costs and outcomes.

Parameter varied from base case analysis	Revised parameter estimate (95% confidence interval)	Revised parameter distribution[a]	Mean decrease with user-testing (95% credible interval)			Incremental cost-saving		Net monetary benefit[b]	
			Moderate-severe pADEs	All pADEs	QALY decrements	Mean ICS (95% credible interval), £	Probability user-testing cost-saving	Mean NMB (95% credible interval), £	Probability user-testing cost-effective
**None – base case (for comparison)**	-	-	157 (−13 to 363)	411 (210 to 675)	147.5 (-24.9 to 406.1)	240,943 (43,527 to 491,576)	0.99	3,190,064 (−£346,709 to £8,480,665)	0.96
**Time horizon**	1 year	Deterministic	31 (−3 to 73)	82 (42 to 136)	31.2 (−5.2 to 87.1)	46,648 (3,177 to 101,006)	0.98	669,944 (−74,287 to 1,815,983)	0.96
**Time horizon**	10 years	Deterministic	315 (−32 to 728)	823 (420 to 1,356)	265.8 (−46.5 to 742.3)	449,553 (79,168 to 911,049)	0.99	5,766,532 (−612,727 to 15,470,264)	0.96
**Error type and error severity probabilities include the possibility of multiple errors per dose**	See Tables F to H		444 (153 to 817)	844 (445 to 1,373)	156.8 (−17.4 to 417.7)	582,900 (235,844 to 1,041,315)	1.00	3,717,908 (91,508 to 9,187,074)	0.98

Parameter varied from base case analysis	Revised parameter estimate (95% confidence interval)	Revised parameter distribution[a]	Mean decrease with user-testing (95% credible interval)			Incremental cost-saving		Net monetary benefit[b]	
			Moderate-severe pADEs	All pADEs	QALY decrements	Mean ICS (95% credible interval), £	Probability user-testing cost-saving	Mean NMB (95% credible interval), £	Probability user-testing cost-effective
**Undetected error frequency with current guidelines reduced to 32%, with unchanged relative reduction in error frequency after user-testing**	See Tables I to L		80 (−77 to 252)	208 (36 to 421)	75.4 (−64.7 to 269.9)	127,725 (−57,148 to 335,690)	0.92	1,636,052 (−1,253,565 to 5,611,961)	0.88
**Undetected error frequency with current guidelines reduced to 32% and relative effect of user-testing halved**	See Tables I to L		51 (−110 to 223)	119 (−49 to 316)	40.6 (−125.9 to 237.5)	79,593 (−108,023 to 280,769)	0.81	892,882 (−2,526,604 to 4,916,336)	0.71

Parameter varied from base case analysis	Revised parameter estimate (95% confidence interval)	Revised parameter distribution[a]	Mean decrease with user-testing (95% credible interval)			Incremental cost-saving		Net monetary benefit[b]	
			Moderate-severe pADEs	All pADEs	QALY decrements	Mean ICS (95% credible interval), £	Probability user-testing cost-saving	Mean NMB (95% credible interval), £	Probability user-testing cost-effective
**Decreased probability an error is undetected prior to administration**	0.100 (0.085 to 0.119)	Log-normal (-2.30,0.086)	20 (−2 to 47)	53 (27 to 88)	18.8 (−3.6 to 52.9)	41,485 (12,199 to 76,494)	1.00	417,865 (−42,470 to 1,113,390)	0.96
**Increased probability an error is undetected prior to administration[c]**	1.0	Deterministic	202 (−18 to 466)	529 (277 to 859)	187.6 (−35.6 to 520.3)	305,915 (51,596 to 626,265)	0.99	4,058,613 (−517,712 to 10,874,000)	0.96
**Increased probability an undetected error causes no harm**	0.992 (0.980 to 1.000)	Beta (238,2)	14 (−1 to 49)	36 (4 to 104)	12.9 (−1.7 to 51.3)	30,229 (5,771 to 78,632)	1.00	287,738 (−11,524 to 1,085,495)	0.97
**Decreased probability an undetected error causes no harm**	0.750 (0.695 to 0.805)	Beta (180,60)	411 (−46 to 901)	1,076 (609 to 1,628)	376.1 (−73.8 to 1,023.5)	612,644 (88,557 to 1,198,633)	0.99	8,134,158 (−1,073,749 to 21,366,371)	0.96

Parameter varied from base case analysis	Revised parameter estimate (95% confidence interval)	Revised parameter distribution[a]	Mean decrease with user-testing (95% credible interval)			Incremental cost-saving		Net monetary benefit[b]	
			Moderate-severe pADEs	All pADEs	QALY decrements	Mean ICS (95% credible interval), £	Probability user-testing cost-saving	Mean NMB (95% credible interval), £	Probability user-testing cost-effective
**Number of doses of intravenous voriconazole administered using IMG per annum**	20,000	Deterministic	787 (−80 to 1,822)	2,057 (1,039 to 3,402)	730.5 (−133.4 to 2,041.3)	1,231,112 (224,306 to 2,508,969)	0.99	15,840,983 (−1,816,590 to 42,701,213)	0.96
**NMB calculated using original Karnon method[d]**	See Table O		158 (−13 to 364)	412 (211 to 681)	-	241,642 (41,195 to 493,055)	0.99	3,660,495 (−164,858 to 10,930,365)[d]	0.97[d]
**QALY decrements following a medication error halved**	See Table P		157 (−17 to 362)	412 (210 to 685)	72.5 (−11.5 to 201.4)	240,963 (41,129 to 492,222)	0.99	1,702,026 (−91,627 to 4,392,120)	0.97
**QALY decrements following a medication error doubled**	See Table P		158 (−14 to 364)	412 (211 to 680)	293.6 (−48.9 to 824.8)	241,768 (42,241 to 493,461)	0.99	6,114,021 (−824,387 to 16,858,756)	0.96

Parameter varied from base case analysis	Revised parameter estimate (95% confidence interval)	Revised parameter distribution[a]	Mean decrease with user-testing (95% credible interval)			Incremental cost-saving		Net monetary benefit[b]	
			Moderate-severe pADEs	All pADEs	QALY decrements	Mean ICS (95% credible interval), £	Probability user-testing cost-saving	Mean NMB (95% credible interval), £	Probability user-testing cost-effective
**Mean medication error costs reduced to 25th percentile of logarithmic cost range**	See Table Q		159 (−13 to 366)	413 (210 to 685)	147.0 (−25.8 to 407.7)	220,143 (36,399 to 450,446)	0.99	3,160,431 (−366,859 to 8,465,831)	0.96
**Mean medication error costs increased to 75th percentile of logarithmic cost range**	See Table Q		158 (−17 to 367)	412 (209 to 684)	146.0 (−25.6 to 406.0)	269,906 (49,129 to 549,054)	0.99	3,190,234 (−311,632 to 8,526,333)	0.96
**Uniform distribution for QALY decrements following a medication error**	See Table P		157 (−15 to 365)	411 (208 to 680)	144.7 (−22.8 to 453.6)	240,330 (40,745 to 493,261)	0.99	3,135,059 (−300,025 to 9,408,090)	0.96

[a]For normal and log-normal distributions, parameters quoted are (mean, standard error).

[b]Willingness-to-pay threshold = £20,000 per QALY

[c]To simulate hospitals which do not have a double-checking policy

[d]NMB calculated using original Karnon method, based on monetary expression of QoL reduction following an error, derived by combining NHS litigation costs, hypothetical QALY decrements and willingness-to-pay thresholds of £20,000 and £30,000 per QALY [7]. Therefore, QALY decrements were not calculated during this sensitivity analysis.

ICS = incremental cost-saving; IMG = Injectable Medicines Guide; NMB = net monetary benefit; pADE = preventable adverse drug event, QALY = quality adjusted life year; QoL = quality of life

The number of doses of voriconazole administered using the IMG per annum

We estimated the number of doses of voriconazole administered using the IMG per annum to convert the probability of each outcome of the decision tree (Figure 1) into an estimated number of patients.

The IMG guide for voriconazole was downloaded 4,130 times from April 2018 to March 2019 (Robin Burfield, NHS Wales Informatics Service personal communication, 30th July 2019), but as guides can be printed, one download may be used several times. We used Freedom of Information legislation to request the number of doses of intravenous voriconazole administered and the number of vials of intravenous voriconazole used in each NHS hospital organisation that used the IMG during this time. Information on the number of doses was not available from every organisation and the dose of voriconazole is variable, so a single dose may require one or more vials. Therefore, where information on the number of doses of intravenous voriconazole administered was unavailable, we used linear regression to estimate the number of doses administered from the number of vials used.

A total of 114 of 121 hospital organisations provided information; 39 provided the number of doses. Linear regression using Equation 1 estimated that β=0.590 (95% confidence interval: 0.561-0.6219).

$$Number\ of\ doses = \beta(number\ of\ vials)$$

Equation 1

Using this estimate of β and Equation 1, we estimated that 22,980 doses (95% confidence interval: 22,122 to 23,827) of intravenous voriconazole were given in hospitals that used the IMG from April 2018 to March 2019.

Derivation of model inputs for medication error costs and QALY decrements

Karnon *et al.* described the costs to the health system of treating the adverse effects of a pADE (including increased hospital length of stay and extra tests and treatments) or correcting a detected medication error [8]. We included these costs in the calculation of both incremental cost saving (ICS) and net monetary benefit (NMB). Karnon *et al.* also used monetary terms to express the quality of life reduction following minor, moderate and severe pADEs. Karnon *et al.* derived these costs by combining NHS litigation costs with hypothetical QALY decrement ranges following a pADE based on limited data and discussion within the research team, using willingness-to-pay thresholds of £20,000 and £30,000 per QALY [7]. In line with more recent approaches to enable the use of cost-effectiveness acceptability curves, our base case analysis used only the Karnon *et al.* QALY decrement ranges in the calculation of NMB (Table N). However, a sensitivity analysis employed the original quality of life costs derived by Karnon *et al.* (Table O of Online Resource). Given the high levels of uncertainty about the hypothetical QALY decrements, additional sensitivity analyses considered doubled and halved QALY decrements (Table P of Online Resource).

We uprated costs to 2018 values using the Hospital and Community Health Services Index (Table N) [9]. We fitted log-normal distributions to the cost ranges, by assuming log-cost was normally distributed with the mean of each distribution set as the midpoint of logarithmic cost range (Table N). We fitted normal distributions to the QALY decrement ranges, with the mean of each distribution set as the midpoint of the range (Table N). For both types of distribution, we assumed that the distance between the upper and lower limits of the cost or QALY ranges represented six standard deviations (i.e. contained 99.7% of the data) and during probabilistic analysis, outputs from these distributions were constrained to the original cost or QALY ranges. Sensitivity analyses considered higher and lower mean cost values (Table Q of Online Resource) and uniform distributions for QALY decrements (Table P of Online Resource).

Table N: Summary of model inputs for medication error costs and QALY decrements for the base case analysis.

Input parameter	Upper and lower limits from Karnon *et al.* [8]	Estimate (95% confidence interval)	Distribution (mean, SD)
Cost of an error detected before administration	£0.01-8.00[a]	£0.27 (0.03 to 2.39)	Log-normal (−1.29, 1.10)
Treatment cost for a minor pADE	£81-188[a]	£124 (94 to 163)	Log-normal (4.82, 0.14)
Treatment cost for a moderate pADE	£1,015-1,544[a]	£1,252 (1092 to 1436)	Log-normal (7.13, 0.07)
Treatment cost for a severe pADE	£1,544-2,206[a]	£1,846 (1643 to 2074)	Log-normal (7.52, 0.06)
QALY decrement following a minor pADE	0.0008-0.0077	0.004 (0.002 to 0.006)	Normal (0.004, 0.0011)
QALY decrement following a moderate pADE	0.0077-0.0614	0.035 (0.017 to 0.052)	Normal (0.035, 0.0089)
QALY decrement following a severe pADE	1.00 to 6.00	3.50 (1.87 to 5.13)	Normal (3.50, 0.83)

[a]Uprated to 2018 values using the Hospital and Community Health Services Index [9].

NHS = National Health Service, pADE = preventable adverse drug event; QALY = quality adjusted life year; SD = standard deviation

Table O: Summary of additional model inputs for medication error costs for the sensitivity analysis which used the original method of calculating net monetary benefit employed by Karnon et al [8]. These 'quality of life costs' replaced 'QALY decrements valued at a willingness-to-pay threshold of £20,000 per QALY' in the calculation of net monetary benefit. Karnon et al. derived them by combining NHS litigation costs with hypothetical QALY decrement ranges following a medication error derived from limited data and discussion within the research team, using willingness-to-pay thresholds of £20,000 and £30,000 per QALY. These were the same QALY decrement ranges employed in the base case analysis of the present study.

**Type of cost**	**Uprated cost range from Karnon *et al.*, £[a]**	**Estimate (95% confidence interval), £**	**Distribution (mean, standard deviation)**
**QoL reduction following a minor pADE**	16-2,445	198 (38-1023)	Log-normal (5.29, 0.84)
**QoL reduction following a moderate pADE**	153-16,598	1,594 (345-7,366)	Log-normal (7.37, 0.78)
**QoL reduction following a severe pADE**	20,000-250,683	70,807 (31,000-161,729)	Log-normal (11.17, 0.42)

[a]Upper limits from Karnon *et al*. [8] uprated to 2018 values using the Hospital and Community Health Services Index [9]. The lower limits were not uprated, as Karnon *et al*. based them on estimated QALY decrements and the NICE cost-effectiveness threshold, which has remained unchanged.

NICE = National Institute for Health and Care Excellence; pADE = preventable adverse drug event; QALY = quality adjusted life year; QoL = quality of life

Table P: model inputs for QALY decrement sensitivity analyses

	**Minor harm**		**Moderate harm**		**Severe harm**	
	**Revised parameter estimate (95% confidence interval)**	**Revised parameter distribution**	**Revised parameter estimate (95% confidence interval)**	**Revised parameter distribution**	**Revised parameter estimate (95% confidence interval)**	**Revised parameter distribution**
**QALY decrements following a medication error halved**	0.002 (0.001 to 0.003	Normal (0.002, 0.0006)[a]	0.017 (0.008 to 0.026)	Normal (0.017, 0.0045)[a]	1.75 (0.93 to 2.57)	Normal (1.75, 0.42)[a]
**QALY decrements following a medication error doubled**	0.008 (0.004 to 0.013)	Normal (0.008, 0.002)[a]	0.069 (0.034 to 0.104)	Normal (0.069, 0.018)[a]	7.00 (3.73 to 10.27)	Normal (7.00, 1.67)[a]
**Uniform distribution for QALY decrements following a medication error**	0.0040 (0.0010 to 0.0075)	Uniform within limits: 0.0008 to 0.0077	0.035 (0.009 to 0.060)	Uniform within limits: 0.0077 to 0.0614	3.50 (1.13 to 5.88)	Uniform within limits: 1.00 to 6.00

[a]Parameters shown are (mean, standard deviation).

Table Q: Summary of model inputs for medication error costs for the sensitivity analyses.

Type of cost	Uprated cost range from Karnon *et al.*, £[a]	Mean cost = 25th percentile of logarithmic cost range		Mean cost = 75th percentile of logarithmic cost range	
		Estimate (95% confidence interval), £	Distribution (mean, standard deviation)	Estimate (95% confidence interval), £	Distribution (mean, standard deviation)
**Cost of an error detected before administration**	0.01 to 8.00	0.05 (0.01 to 0.46)	Log-normal (−2.95, 1.10)	1.44 (0.17 to 12.50)	Log-normal (0.36, 1.10)
**Treatment cost for a minor pADE**	81 to 188	100 (76 to 132)	Log-normal (4.61, 0.14)	153 (116 to 200)	Log-normal (5.03, 0.14)
**Treatment cost for a moderate pADE**	1,015 to 1,544	1127 (983 to 1293)	Log-normal (7.03, 0.07)	1,391 (1,212 to 1,595)	Log-normal (7.24, 0.07)
**Treatment cost for a severe pADE**	1,544 to 2,206	1688 (1503 to 1897)	Log-normal (7.43, 0.06)	2,018 (1,796 to 2,267)	Log-normal (7.61, 0.06)

[a]Upper and lower limits from Karnon *et al*.[8] uprated to 2018 values using the Hospital and Community Health Services Index [9].

NHS = National Health Service, pADE = preventable adverse drug event; QoL = Quality of Life

Online Resource References

1. Jones MD, McGrogan A, Raynor DKT, Watson MW, Franklin BD. User-testing guidelines to improve the safety of intravenous medicines administration: a randomised in-situ simulation study. BMJ Qual Saf. 2020;30:17-26.
2. Koyama AK, Maddox CS, Li L, Bucknall T, Westbrook JI. Effectiveness of double checking to reduce medication administration errors: a systematic review. BMJ Qual Saf. 2019 Aug 7;29(595-603).
3. Douglass AM, Elder J, Watson R, Kallay T, Kirsh D, Robb WG, et al. A randomized controlled trial on the effect of a double check on the detection of medication errors. Annals of Emergency Medicine. 2018 Jan;71(1):74-82 e1.
4. Modic MB, Albert NM, Sun Z. Does an insulin double-checking procedure improve patient safety? J Nurs Adm. 2016;46(3):154-60.
5. Kruse H, Johnson A, O'Connell D, Clarke T. Administering no-restricted medicines in hospitals: the implications and cost of using two nurses. Australian Clinical Review. 1992;12(77-83).
6. Blandford A, Furniss D, Galal-Edeen GH, Chumbley G, Wei L, Mayer A, et al. Intravenous infusion practices across England and their impact on patient safety: a mixed-methods observational study. Health Services and Delivery Research. 2020;8(7):1-116.
7. Karnon J, McIntosh A, Bath P, Dean J, Hutchinson A, Oakley J, et al. A prospective hazard and improvement analysis of medication errors in a UK secondary care setting2007.
8. Karnon J, McIntosh A, Dean J, Bath P, Hutchinson A, Oakley J, et al. Modelling the expected net benefits of interventions to reduce the burden of medication errors. J Health Serv Res Policy. 2008 Apr;13(2):85-91.
9. Curtis L, Burns A. Unit Costs of Health and Social Care 2018: Personal Social Services Research Unit, University of Kent, Canterbury; 2018.
